# Patients’ perspectives towards biologic dose reduction in psoriasis: a qualitative study

**DOI:** 10.1007/s00403-023-02566-w

**Published:** 2023-02-23

**Authors:** L. S. van der Schoot, L. M. Verhoef, I. van Ee, F. P. A. H. van Oort, A. H. Pieterse, M. M. B. Seyger, E. M. G. J. de Jong, J. M. P. A. van den Reek

**Affiliations:** 1 Department of Dermatology, Radboud University Medical Center, Nijmegen, The Netherlands; 2 Radboud Institute for Health Sciences, Radboud University Medical Center, Nijmegen, The Netherlands; 3 Department of Rheumatology, Sint Maartenskliniek, Nijmegen, The Netherlands; 4 Psoriasispatiënten Nederland, Dutch National Psoriasis Patient Association, Nijkerk, The Netherlands; 5 Radboud University, Nijmegen, The Netherlands

**Keywords:** Biologics, Psoriasis, Patients, Qualitative research

## Abstract

Dose reduction of biologics for psoriasis could contribute to more efficient use of these expensive medicines. Evidence on opinions of patients with psoriasis regarding dose reduction is sparse. The objective of this study was therefore to explore patients’ perspectives towards dose reduction of biologics for psoriasis. A qualitative study was conducted, comprising semi-structured interviews with 15 patients with psoriasis with different characteristics and treatment experiences. Interviews were analyzed by inductive thematic analysis. Perceived benefits of biologic dose reduction according to patients were minimizing medication use, lowering risks of adverse effects and lowering societal healthcare costs. Patients reported to have experienced a large impact of their psoriasis, and expressed concerns about loss of disease control due to dose reduction. Fast access to flare treatment and adequate monitoring of disease activity were among reported preconditions. According to patients, they should have confidence in dose reduction effects and should be willing to change their effective treatment. Moreover, addressing information needs and involvement in decision-making were deemed important among patients. In conclusion, addressing patients’ concerns, fulfilling information needs, providing the possibility of resuming standard dose, and involving patients in decision-making are important according to patients with psoriasis when considering biologic dose reduction.

## Introduction

Biologics have allowed patients with psoriasis to achieve adequate disease control, or even complete clearance [2]. The possibility of achieving good treatment responses now poses the question how these patients should be treated over the long term. Sustainable use of the expensive biologics is important as healthcare costs are increasing and access to biologics is unequal on a global scale. Dose reduction (DR) of biologics by means of injection interval prolongation for patients with stable low disease activity enables more efficient use of biologics with decreasing healthcare costs [3, 4, 17].

DR is already performed in daily practice but not on a standard basis [6, 22]. Striving for standardization is however important: it leads to consistent and safe practice, and better uptake in routine care. For adoption of DR into practice, insight in factors which hamper or facilitate implementation is needed. Barriers to implementation of DR may arise at the patients’ level [10, 11]. We previously showed that ‘fear for psoriasis flares’ was the most important reason among patients with psoriasis for unwillingness to start DR in a daily practice evaluation study on DR of adalimumab, etanercept or ustekinumab [5]. In rheumatology, several qualitative studies reported that fear of relapse was a main concern among patients with inflammatory arthritis when considering biologic DR [23]. Nevertheless, patients were willing to try DR after receiving information on DR, and if increasing the dose was possible when deemed necessary [12]. Currently, in-depth explorations of opinions of patients with psoriasis toward DR are lacking.

The aim of this study was to explore perspectives of patients with psoriasis regarding biologic DR. Results will inform healthcare providers on what is important for patients within the context of DR and could provide a solid basis for shared decision-making.

## Materials and methods

### Study design

A cross-sectional qualitative study was performed consisting of semi-structured interviews. A qualitative design was considered most suitable for broadly exploring patients’ perspectives [7]. Reporting of this study followed the Standards for Reporting Qualitative Research [18].

### Participants

Adult patients with psoriasis treated with biologics at the department of Dermatology, Radboudumc, Nijmegen, the Netherlands, were recruited from the prospective BioCAPTURE cohort [21]. Purposive sampling was used to obtain a variety of patients regarding age, sex, type of biologic, treatment duration, treatment history, and experience with DR, to get insight into a broad range of experiences. Participants were not necessarily candidates for DR yet. Ethical approval was waived by the medical ethical committee Arnhem-Nijmegen (2021-12967). All participants provided written informed consent.

### Data collection

A semi-structured interview guide was developed by the research team (LS, LV, JR, EJ), and addressed experiences with biologic treatment and DR, opinions and beliefs regarding DR, willingness to try DR, perceived opinions of people in patients’ environment, preconditions for biologic DR, and information needs. Patient representatives from the national patients’ association (IE, FO) reviewed the interview guide.

Interviews were conducted between September 2021 and March 2022 by one researcher (LS). This researcher/physician (LS) had no long-term treatment relationship with participants, but conducted consultations with some participants in the past 6 months. Recruitment of patients ended when data saturation was reached (no new subthemes emerged from the last three interviews). Interviews were held by telephone, were audio-recorded and transcribed verbatim. No formal member check was performed, but a summary was presented to the participant at the end of the interview to check for accuracy. Patient and treatment characteristics were collected from BioCAPTURE.

### Data analysis

Transcribed interviews were analyzed by inductive thematic analysis using ATLAS.ti software. Thematic analysis comprises a flexible approach for identifying themes, without trying to fit data into any predetermined category [8]. Repeated transcript reading was conducted to maximize data familiarity. Analytical rigor was sought using multiple coders (investigator triangulation) [18]: two researchers (LS, LV) coded the first three transcripts independently (open coding), resulting in a list of initial codes. Based on initial codes, next transcripts were systematically coded (axial coding) by one researcher (LS), and reviewed by another researcher (LV). Differences were discussed until consensus was reached. Newly identified themes were added to the codebook, using an inductive approach. Analysis was concurrent with data collection, to explore emerging themes further on in later interviews. When necessary, the interview guide was adapted. Constant comparison was applied throughout the whole process of data analysis, by comparison of emerging themes with new data [18]. Results and saturation were discussed during the process. Analysis resulted in (sub)themes that play a role for patients with psoriasis regarding biologic DR. Corresponding illustrative quotes were selected from the interviews, and were translated into English by a professional translation service. Results were revised based on discussion with key researches (LS, LV, JR, and EJ). Patient representatives (IE, FO, and AP) reviewed results to check whether results were formulated in a way compatible to the patient perspective.

Patient characteristics were summarized using SPSS Statistics version 25 (IBM Corp., Armonk, NY).

## Results

Patient characteristics are summarized in Table 1. Among participants, differences in current biologic treatment, number of previous biologics used and experience with DR were present. Saturation was achieved after 15 completed interviews. Mean duration of interviews was 28 min. After analyzing the first three interviews, minor changes were made to the interview guide including addition of questions regarding psoriatic arthritis (PsA), and preferences for timing and channeling of information.

**Table 1:** Baseline patient characteristics

Characteristic	Total N = 15
Sex (female)	6 (40)
Age (years)^a^	43 (28–75)
Disease duration until present (years)^a^	18 (7–47)
Psoriatic arthritis (yes)	5 (33.3)
Previous biological treatment	
Yes	9 (60)
No	6 (40)
Number of previous biologics used^a^	1 (0–6)
Current biologic	
Adalimumab	4 (26.7)
Etanercept	2 (13.3)
Ustekinumab	3 (20.0)
Secukinumab	1 (6.7)
Ixekizumab	2 (13.3)
Brodalumab	1 (6.7)
Risankizumab	2 (13.3)
Treatment duration of current biologic (years)^a^	4 (0.5–14)
Experience with biologic DR^b^	
Yes, successful	4 (26.7)
Yes, unsuccessful	4 (26.7)
No	7 (46.7)

Data are presented as *N* (%) unless otherwise indicated

^a^Median (range)

^b^Successful dose reduction was defined as use of a lower dose and maintenance of low disease activity (PASI ≤ 5). Abbreviations: DR: dose reduction

**Fig. 1:**
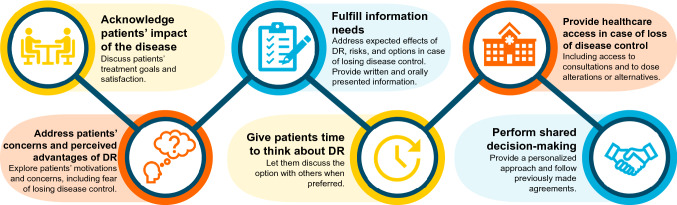
Summary of clinical implications based on identified (sub)themes arising from the interviews

In total, seven main themes were identified: disease control, attitudes towards medication and DR, healthcare access and organizational aspects, cost reduction, information needs, social aspects, and decision-making. Corresponding subthemes are described below. An overview of (sub)themes and illustrative quotes is presented in Table 2. Summarized clinical implications based on (sub)themes arising from the interviews are graphically presented in Fig. 1.

**Table 2:** Identified (sub)themes with illustrative quotes of patient perspectives towards biologic dose reduction in psoriasis

Theme	Subtheme	Quotes
Disease control	Impact of psoriatic disease	‘If the patches get worse, it will once again affect how I feel and the things I struggle with. All my old symptoms will return, and I will constantly itch in annoying places. It will show up in my face, and I won’t be able to style my hair the way I like, because I will be covered in it. And it will hurt when I have sex.’ [P6]
	Effort to reach low disease activity	‘Look, you know where you’ve come from and that this isn’t something you want to go back to, and I think that most people have had quite a long journey getting here. Before you get to the biologicals, you’ve usually tried two or three other things. Probably, none of them helped or not enough, and then the biological is this great solution, so clearly, you’re not willing to lose it again.’ [P15]
	Fear of disease flare	‘It’s not that I’m completely negative about it, only that if it gets worse, if the skin condition makes it difficult to move… Now, I’m not afraid to take a shower with the other guys. If more patches appear, it will be harder to move, so I’ll no longer be able to play football one hour a week, or cycle with my child, and that certainly won’t make my life easier.’ [P12]
	Treatment goals	‘I find it very important that my skin is healthy, and this is ultimately why I do it. If it stays the way it is for a year or so, without getting better or worse, I wouldn’t mind trying to reduce the dose. But if I’m not fully satisfied, then I wouldn’t agree to a lower dose. In fact, I’d probably be worried about whether the full dose is effective enough.’ [P8]
Attitudes towards medication and DR	Attitude towards possible adverse effects	‘On the other hand, it works really well and I’m not experiencing any side effects, so I don’t necessarily feel the need to reduce the dose.’ [P6]
	Willingness to change effective treatment	‘We have finally found something that works well for me, so I’d rather not mess with it. When something works, don’t change it; that’s how I feel about it intuitively.’ [P8] ‘Well, I would like to take the gamble. Because clearly, this can only be a win–win situation. It may be that your body responds well to the change, that you can handle a lower dose, which is great. If you don’t try it, you’ll never know. And if you find that you have to increase the dose again, at least you will have given it a try.’ [P5]
	Use of concomitant topical treatment	‘I’ve said it very clearly: I don’t want to apply anymore of those ointments. All that ointments; I hardly had any skin left. And it’s disgusting, fatty stuff. It made everything dirty: my clothes, my sofa, my bed, etc. I couldn’t even hug my own children. So I’m completely done with those ointments; I don’t want to have anything more to do with them.’ [P9] ‘I’m now supplementing with ointments, so I hope to see the effects better in a few weeks. If not, then I won’t wait, but I’ll switch right back to injections every 14 days.’ [P5]
	Confidence in DR	‘It’s not as if you’re suddenly completely covered in it, but it does take hold quite fast. Once it flares up, things can go fast. But I also noticed with the medication, that it starts working pretty quickly, so I expect it to take effect quickly too, which is why I’m not scared at all.’ [P3] ‘If the injection interval is extended now, I don’t know what will happen. But as long as I don’t return to the old level, and I remain at the level I’m at now, where I can go about my life, and sit down with my child on the floor, play with Lego, etc., that’s worth more to me than trying to extend the period.’ [P12]
	Practical use of the biologic	‘I don’t actually care that much how often it is. You just put it on your calendar, and make sure you’ve got enough medication in stock.’ [P5] ‘It’s a question of ease, I think. The lower the dose, the less work for me. Now I’ve got one medicine that needs to be administered every twelve weeks, which isn’t much at all. But I’ve also had medicines in the past that had to be administered every two weeks. Well, it would be great if I could reduce that to once every four weeks.’ [P8]
	Minimizing medication use	‘It’s about the health aspect, right? These are biologicals, it’s not just a simple medicine they’re administering. I do try to think of my health; everyone does. I know it’s a tough medicine; I’m fully aware of it. The less of it I need, the better for my body, as long as my skin stays in good shape, that is.’ [P5]
Healthcare access and organizational aspects	Access to treatment in case of disease flares	‘If you try it once and find it doesn’t work, or the symptoms get worse fast, and you can simply return to your previous dose, if that’s something that is easy to arrange, then I wouldn’t necessarily be against it.’ [P6] ‘At the start of the dose reduction track, there should already be a back-up plan ready. (…) Once that can be implemented immediately, without first having to wait one, two, three, or four months, while the symptoms are getting worse. Because this is a cumulative process; it starts with a small patch, and before you know it, your whole leg is covered in it.’ [P12]
	Access to the outpatient clinic	‘The only thing that matters is that it should be possible to get an appointment if the symptoms return, or if I have questions or concerns, that I can call them. It should be possible at any time to plan in an interim appointment.’ [P9] ‘Maybe the monitoring could be done in the form of an app. (…) For example staying in touch via an app in the early phase of reducing the dose. That way, you don’t necessarily need to see a doctor immediately; you can wait until the doctor has time. It would make things much easier, and I think it would work quite well.’ [P3]
	Importance of monitoring	‘If we have more frequent check-ups and someone checks up on me on a regular basis, and this doesn’t necessarily have to be a physical check-up, it could be contact by telephone, for example every six weeks. I think that, as a patient, I am quite capable of noticing whether things are improving or not. Because in this case, we’re talking about a skin disease, and it’s quite easy to see whether your skin is doing well or not. But maybe there could also be some blood tests, that kind of thing. If those kinds of tests are run more frequently at first, that would give me the feeling that the situation is being monitored adequately.’ [P8]
	Barriers at the healthcare providers' level	‘I understand that they don’t go through my entire file in depth, and I don’t see the same dermatologist every time. So within the short time that this person is treating me, they have to go through my whole file, and then it’s easiest to say: It’s going well, great, let’s keep going!’ [P1]
Cost reduction	Contribution to lowering costs of the national healthcare budget	‘I know that this medicine is also very expensive, so why would I want to keep going no matter what. Especially if I can keep my condition stable with less. Maybe this means that other people can also get better help, and this might in turn keep the costs of health insurance lower for longer.’ [P3]
	Availability of biologics to more patients	‘I think that lowering the dose means that you can inject two people for the price of one. So I think in this way you can help more people, if you make biologicals more easily accessible. I think it would help a lot more people.’ [P9]
Information needs	Content of information about DR	‘What I’m mostly curious about is what previous research has to say about it. Because if this is offered to me as a patient, I’m assuming it has been tried before, at least to some extent. And that there are known results. That would help me to know better where I stand, and what I should also take into account.’ [P8]
	Preferences for timing and channel of information	‘I would like to get it from a doctor. Someone who has done research on it, or at least knows a lot about it.’ [P14] ‘When I’m sitting in one of your treatment rooms, and you sit opposite me, I’m able to process the information quickly, and figure out what it means for me. But that’s not the case for everyone, which is why having a bit more time might be useful. And maybe also providing some kind of information, so I can read through it again at home. This is always useful, as much information as possible. It also depends a bit on what the risks are. Look, if nine people out of ten benefit from it, or have no negative effects from the lower dose, then I’d say, go ahead. And in that case, I don’t really need much more information than that.’ [P8]
Social aspects	What others in patients' environment think of DR	‘My wife, of course. I’d certainly like to discuss it with her. Clearly, she also knows what it was like when I was suffering from it, and how ashamed I was. Plus, she might have questions about the future, and maybe she can see something that I haven’t considered at all.’ [P7] ‘As far as I’m concerned, this is my decision. It’s my life, my body, my decision. I may tell people about it, but as I said, only for information purposes.’ [P9]
	Healthcare provider-patient relationship	‘Maybe that last bit, about being heard. If I have the feeling that the effect is diminishing, and my symptoms are returning, I want to be taken seriously, and not end up in a discussion with the doctor about what I find acceptable versus what they find acceptable.’ [P6]
Decision-making	Expertise of the treating healthcare provider	‘Just to be able to talk about it to the doctor, because this is all new to me, and you people have some experience with it. I don’t have any experience with it, and I don’t know anyone who does, you see. Which is why it’s important to have a doctor who can say: listen, this is normal, and this isn’t.’ [P2] ‘I’m open to it, and I trust the doctors I’m talking to. I also feel that my opinion matters, so I completely trust it, and if it doesn’t work, I can always go back, of course.’ [P5]
	Personalized approach	‘When I first came in, this man asked me: How is it going? Fine. That first time, I rolled up my sleeves to show him my skin. But he said: No, I’m asking you how you are, not how your skin is. That really struck me: this is a human being, across from you. That’s what I find important. Knowing that there is a human being in front of you, having an experience; not a number. Not: this is number six today, and I have to make sure they get off their medication. No, there is a human being there, and every human being is different. This is something I find very important.’ [P13] ‘I don’t think I can recommend it to other people; I can only speak from my own experience. If people want to do it, that’s fine, but I just want to tell them: keep an eye on it. By the way, I don’t think any two cases of psoriasis are identical.’ [P11]
	Decision-making process	‘I’m pretty motivated to lower the dose, but I do feel strongly that this is my decision. And that’s what I find really important.’ [P9] ‘Well, I think it would be good if it started with a face-to-face talk, and then some information on paper, so you could read about it and think it through. That you would have time to think about it, instead of having to decide on the spot.’ [P6]

### Disease control

#### Impact of psoriatic disease

Disease control was brought up as an important issue. Patients reported to have experienced a large physical and/or psychosocial impact of their psoriasis, which also involved feelings of shame. Having psoriatic disease also had an impact on work and daily functioning. Causing itch, pain and scaling, psoriasis had a large impact on patients’ wellbeing. In case of concomitant PsA, joint symptoms had a major impact on patients’ lives as well. In contrast, patients mentioned that being free of psoriatic disease provided a feeling of freedom. As such, patients did not want to go back to the days when they had severe psoriasis. Participants expressed that healthcare providers should pay attention to the (past) impact of the disease within the context of DR.

#### Effort to reach low disease activity

Patients valued the option of the highly effective biologics. Participants reported to have used different treatment modalities over time, before qualifying for biologics. The effort to reach a state of low disease activity was therefore an identified barrier to DR. Initiation of DR after a long period of biologic treatment was reported as a factor to overcome this possible barrier, as this could gain patients’ trust in DR effects and might prevent loss of effectiveness according to patients.

#### Fear of disease flares

Related to the past experiences of having severe psoriasis, fear of disease worsening was identified as an important theme among participants. It was reported that the risk of disease flares could result in unwillingness to start DR. Patients experienced with DR noted that concerns about disease flares may disappear after perceived maintained treatment effectiveness despite reducing the dose.

#### Treatment goals

Adequate disease control can be important for patients when starting or continuing DR. However, definitions of adequate disease control and the ideal duration of having good treatment effects before starting DR can differ between patients. The amount of psoriasis lesions and location of lesions could contribute to perceived disease activity. Treatment goals can change over time, depending on patients' life course and previous (un)successful treatment experiences. Hence, it was suggested that patients’ goals should be addressed by healthcare providers. Although the accepted level of disease activity might differ between patients, participants mentioned that DR should preferably be initiated after adequate disease control is reached and maintained for a prolonged period.

### Attitudes towards medication and DR

#### Attitude towards possible adverse effects

Having experienced side effects or concerns regarding unknown risks of biologics may act as facilitators for DR. While some participants expressed these concerns, others did not fear the risk of side effects at all. Conversely, not experiencing side effects may act as a barrier for DR, as patients could see no reason why they would change their treatment.

#### Willingness to change effective treatment

Willingness to change effective treatment can play a role when considering DR. Participants expressed that (un)willingness to try DR depends on received information. Moreover, maintaining good treatment effects was deemed important for patients while reducing the dose, and confidence in effectiveness is needed before treatment alterations, according to patients. It was reported that the decision to start with DR could be based on a balance between willingness to try versus risking a flare. For patients experienced with DR, negative or positive experiences with DR could contribute to the willingness of reducing the dose again.

#### Use of concomitant topical treatment

Different views regarding the use of topical treatment were expressed. Some patients preferred to use topicals first before resuming the standard dose in case of increased psoriasis activity following DR. However, patients could also have experienced ineffectiveness of topicals or have negative feelings towards the use of topicals. As such, not having to use topicals again before resuming the standard dose was also mentioned as prerequisite before considering DR.

#### Confidence in DR

Participants expressed that there should be a level of confidence in the DR process. Confidence in DR effects and in reaching adequate treatment responses again after increasing the dose in case of psoriasis worsening were reported facilitators for DR.

#### Practical use of the biologic

As biologics are usually self-injected, administrating less injections was a perceived benefit of DR, specifically for patients who disliked self-injecting. Likewise, less pharmacy visits or less pharmacy delivery moments were expressed as possible advantages of DR. However, for other patients, self-injections were perceived to be convenient and thus less injections will not provide much benefits.

#### Minimizing medication use

Minimizing medication use was an important reported motivation for DR. Participants expressed that medication in general is not good for one’s body and there could be unknown long-term risks. Besides, patients did not want to be dependent of medication. However, biologics are needed for disease control, but the lesser the use, the better.

### Healthcare access and organizational aspects

#### Access to treatment in case of disease flares

The possibility to re-increase the dose in case of psoriasis worsening following DR was a very important condition for patients. Fast access to dose alterations or alternative treatments was deemed essential. In addition, information about alternative treatment options in case of ineffectiveness of resumption of the standard dose should be available at DR start.

#### Access to the outpatient clinic

Patients appreciated the option to contact the outpatient clinic by telephone or digitally in case of questions or increased psoriasis activity, as this could be trust-gaining. After starting DR, a certain level of support should be available, for example through a digital app or a dedicated healthcare provider. This facilitates prompt access to care and generates feelings of being supported.

#### Importance of monitoring

Different opinions related to the frequency and importance of monitoring in the context of DR were expressed. Some patients wanted to have frequent outpatient visits after starting DR, while others did not. It was reported that live visits including skin-checks and blood monitoring could give feelings of being looked after. When starting DR, monitoring disease activity was mentioned as an important factor. More generally, laboratory checks were valued due to possible risks of biologics.

#### Barriers at the healthcare providers’ level

According to patients, possible barriers could lie at the healthcare providers’ level as well. It was indicated that the treating healthcare provider should be aware of DR as treatment option. For them, continuing the standard dose might be the easiest way to go. Additionally, participants suggested that involvement of different healthcare providers might limit application of DR, as the treating clinician should know the patients history to make an estimation of eligibility for DR.

### Cost reduction

#### Contribution to lowering costs of the national healthcare budget

Lowering costs was mentioned as an advantage of DR, as biologics are expensive. However, for individual patients there was no direct financial advantage, as they pay a fixed amount for their healthcare insurance in the Netherlands which covers their treatment costs. Still, it was reported that patients could contribute to lowering societal healthcare costs too. By contrast, patients mentioned that this would not be the case anymore when less expensive alternatives would become available (e.g., biosimilars).

#### Availability of biologics to more patients

Besides contributing to lowering societal healthcare costs, patients expressed the hope that biologics might become available for more patients due to decreased costs resulting from DR.

### Information needs

#### Content of information about DR

Participants emphasized the need for information on DR, including the rationale and evidence, expected effectiveness and potential risks of DR. Study results or previous experiences could help to gain realistic expectations. Furthermore, it was reported that information on treatment options in case of loss of disease control during DR should be provided. Some patients reported that they wanted as much information as possible. However, it was also mentioned that it could differ between patients how much information is needed or preferred to agree to DR.

#### Preferences for timing and channel of information

Participants suggested that information should be presented by the treating healthcare provider during a face-to-face consultation as this promotes direct communication. As such, the healthcare provider can accurately estimate DR eligibility. Written information could add to orally presented information, could help patients to think about DR, and could provide more time for consideration. It was reported that the option of DR could be presented at the start of biologic treatment or alternatively when patients have sustained low disease activity and would be thus eligible for DR.

### Social aspects

#### What others in patients' environment think of DR

Patients were asked about factors that may influence their decision to start DR. Consequently, they reported that discussing the option of DR with relatives or other close contacts is important, as the impact of having psoriasis also affects them. Other people can have other opinions or questions which might aid in the decision-making process. On the other hand, it was mentioned that patients would want to take the decision to start with DR themselves, as it concerns their own body and treatment.

#### Healthcare provider–patient relationship

Participants mentioned that healthcare providers should follow previously made agreements, for example regarding the option to resume the standard or previous dose after DR failure. Likewise, it was deemed important that healthcare providers listen, take patients seriously, and take time, as this is trust-gaining.

### Decision-making

#### Expertise of the treating healthcare provider

A certain level of expertise of the involved healthcare provider with DR could give confidence according to patients. Moreover, some patients rely on advice of their physician. According to patients, physicians’ experience with DR would imply that DR eligibility will be adequately estimated.

#### Personalized approach

As described before, impact of psoriasis and treatment history can differ between patients. Therefore, it was noted that a personalized approach is important when considering DR. Some patients are willing to try DR and others are not, and this should be respected. Moreover, it was reported that both patients’ physical and mental health need to be addressed.

#### Decision-making process

Participants emphasized the need for clear agreements on DR processes. After receiving information, time should be offered to think about DR. Patients mentioned that the healthcare provider should initiate the conversation on biologic DR, but patients should be involved in the actual decision-making. Giving patients a choice is trust-gaining. Participants also reported that in case of psoriasis worsening, the next step should be discussed with the patient and decisions should be made with the patient as well.

## Discussion

This qualitative study explored perspectives of patients with psoriasis towards biologic DR. Inductive thematic analysis of interviews with 15 patients with psoriasis uncovered seven main themes and 23 subthemes that play a role for patients when considering DR. Among concerns of patients was the risk of disease flares due to DR. Fast access to flare treatment and adequate monitoring of disease activity were among reported preconditions. By contrast, motivations for DR could be minimization of medication use, less injections, and a lower risk of adverse effects. Patients valued a good relationship with, and expertise of, their treating healthcare provider. Moreover, addressing information needs together with patient involvement in decision-making were deemed important.

To our best knowledge, this is the first study providing a broad exploration of perspectives of patients with psoriasis on biologic DR. In the context of DR, patients reported fear of disease worsening. This might be related to the reported (past) impact of the disease on patients’ lives, which has been described before [1, 14, 20, 25]. A previous interview study among patients with psoriasis indicated having less impact of psoriasis as an important treatment goal, and the fear that treatment would not be effective (anymore) was reported as well [15]. In line with our results, fear of biologic discontinuation and the struggle to receive biologics were among reported issues by psoriasis patients before [15, 20]. These issues should be addressed by healthcare professionals to reduce patients’ uncertainties [20]. A recent review showed that symptom control, treatment safety, confidence in care, communication with healthcare professionals and costs were among frequently reported patient-relevant outcomes in psoriasis [13]. These findings correspond with our data and indicate that addressing concerns and needs of patients is of great importance when adjusting treatment.

We identified several factors influencing patients’ willingness to try DR. Among patients’ reported barriers was the fear for delayed access to consultations or to the previous dose in case of psoriasis worsening. Some participants reported the importance of monitoring disease activity during consultations, but actual preferences for frequency of monitoring were not provided. Preferences might depend on past experiences with received care and its quantity. Moreover, we believe that DR should be aimed at patients with low disease activity, with the goal to strive for the lowest effective dose. When educating patients and providing healthcare access in case of diminishing treatment effects, patients’ whish for sufficient monitoring could be accommodated.

Previous studies among patients with inflammatory arthritis have found similar patient-reported factors towards biologic DR [9, 12, 16, 23, 24]. Of note, DR strategies for inflammatory arthritis have been incorporated in guidelines and patients might consequently be used to the concept of DR as part of standard care [19]. It has also been suggested in rheumatological literature that by informing patients about possible future DR at treatment start, awareness and confidence could be created [9, 12, 16]. For psoriasis, guidance of patients toward DR could as such be improved with incorporation of DR strategies into clinical practice and treatment guidelines.

This study has several strengths. First, we included a variety of patients with different treatments and experiences with DR, which allowed us to explore relevant perspectives of patients with psoriasis. Second, using a qualitative approach, a comprehensive exploration of patient-relevant factors could be accomplished. Results could contribute to further implementation of DR strategies by incorporating the patient perspective.

Limitations can be found in the study design. Data interpretation could have been influenced by possible framing of questions during interviews, and there might have been other relevant factors of which patients were unaware or which were not mentioned due to social desirability. Due to the qualitative design, interpreting exact numbers and drawing conclusions on most important patient-related opinions was not possible. Reported experiences might be culturally dependent and different findings could be found in other settings. Therefore, replication of this study in other settings would be of added value.

Results of this study show that patients’ impact of their disease and concerns related to DR should be acknowledged when further implementing DR. According to patients, information on and access to flare treatment should be incorporated in DR strategies, as well as information on DR rationale, expected effects and potential risks. Development of clear patient information and incorporation of patient-relevant factors into clinical guidelines on DR could enhance shared decision-making. Standardization of DR strategies and uptake in regular care may gain trust with patients. However, addressing individual treatment goals and satisfaction, and give each patient a choice remains important.

In conclusion, perceived benefits of biologic DR according to patients with psoriasis were minimizing medication use, lowering risks of adverse effects, and lowering societal healthcare costs. However, patients could have concerns related to loss of disease control following DR. Patient interviews indicated that this barrier needs to be addressed by providing adequate information, involving patients in decision-making, offering patients time to think about DR, and providing fast access to healthcare in case of disease flares. By acknowledging these patient-relevant factors when considering DR, further implementation of biologic DR strategies into clinical practice can be facilitated.

## Data Availability

The data that support the findings of this study are available from the corresponding author upon reasonable request.
